# The predictive value of CatLet© angiographic scoring system for long-term prognosis in patients with acute myocardial infarction presenting > 12 h after symptom onset

**DOI:** 10.3389/fcvm.2022.943229

**Published:** 2022-09-21

**Authors:** Heng Wang, Yi He, Jia-Li Fan, Xu Li, Bing-Yuan Zhou, Ting-Bo Jiang, Yong-Ming He

**Affiliations:** Division of Cardiology, The First Affiliated Hospital of Soochow University, Suzhou, China

**Keywords:** angiographic score, acute myocardial infarction, Percutaneous Coronary Intervention, major adverse cardiac events, prognosis

## Abstract

**Background:**

We have recently developed the ***C***oronary ***A***rtery ***T***ree description and ***L***esion ***E***valua***T***ion (CatLet©) angiographic scoring system, which is capable of accounting for the variability in coronary anatomy, and risk-stratifying patients with coronary artery disease. This study aimed to clarify whether the CatLet score had a predictive value for long-term prognosis in patients with acute myocardial infarction (AMI) presenting > 12 h after symptom onset.

**Materials and methods:**

The CatLet score was calculated for 1,018 consecutively enrolled AMI patients, who were divided into 3 groups according to the CatLet score tertiles. The primary endpoint was major adverse cardiac events (MACEs), defined as a composite of myocardial infarction, cardiac death, and ischemia-driven revascularization; secondary endpoints were all-cause death, cardiac death, myocardial infarction, and ischemia-driven revascularization.

**Results:**

The CatLet score was capable of predicting long-term prognosis at a median 4.9-year follow-up alone or after adjustment for risk factors. Multivariable-adjusted hazard ratios (95% CI)/unit higher score were 1.06 (1.05–1.08) for MACEs, 1.05 (1.03–1.07) for all-cause death, 1.06 (1.04–1.09) for cardiac death, 1.06 (1.04–1.08) for myocardial infarction, and 1.06 (1.04–1.08) for revascularization. The univariate model showed good calibration (χ^2^ = 8.25, *P* = 0.4091) and good discrimination (area under ROC curve = 0.7086) for MACEs.

**Conclusion:**

The CatLet score is an independent predictor of long-term clinical outcomes of patients with AMI presenting > 12 h after symptom onset (http://www.chictr.org.cn; Registry Number: ChiCTR2000033730).

## Introduction

A lesion that causes coronary artery flow limitation has been considered as an important prognostic factor (1, 2). The Synergy between Percutaneous Coronary Intervention with Taxus and Cardiac Surgery (SYNTAX) score, an anatomic coronary scoring system, has been developed to grade the severity and complexity of coronary artery disease (CAD) (3). However, the SYNTAX score, solely based on the dichotomized left or right coronary dominance, has failed to adequately describe the variability of coronary anatomy (4, 5). Although the SYNTAX score has been widely used in risk stratification and selection of revascularization strategies, there are studies questioning its predictive value in CAD patients (6–9). We have recently developed a novel ***C***oronary ***A***rtery ***T***ree description and ***L***esion ***E***valua***T***ion (CatLet©) angiographic scoring system, which can be utilized to account for the variability in coronary anatomy, and to risk-stratify patients with CAD (10, 11).

Our prior study showed that the CatLet score better predicted the long-term prognosis among patients who survived AMI presenting at ≤ 12 h and received primary PCI than the SYNTAX score, with a satisfactory inter- or intra-observer reproducibility (11–13). The current study aimed to validate that the CatLet score remained a useful tool for outcome prediction in patients with AMI presenting > 12 h after symptom onset.

## Materials and methods

### Patients

Patients with AMI presenting > 12 h after symptom onset were consecutively enrolled in this study between January 2012 and September 2015. Exclusion criteria included: (a) poor image quality; (b) coronary artery embolism; (c) abnormal coronary anatomy; (d) normal CAG results; and (e) lost to follow-up.

### Scoring method

We have described the CatLet score in detail elsewhere (10–12). In short, it is a new scoring system that attempts to adequately consider coronary variability and risk-stratify patients with coronary artery disease. In the CatLet score, both the left anterior descending artery (LAD) and diagonals (Dx) were classified into three types, and the right coronary artery (RCA) was classified into six types. As a result, a total of 54 (3*3*6) types of coronary circulation pattern were generated. Weighting factors were assigned to each coronary segment according to its subtended myocardial territory. Lesions with diameter stenosis ≥ 50% in vessels ≥ 1.5 mm were scored. For lesions with 50–99% diameter stenosis, we defined the coefficient as 2.0; for lesions with total occlusion, the coefficient was defined as 5.0. Non-occlusive lesions were scored straightforward; in patients whose infarct-related artery is completely occluded, wiring or use of a small balloon is helpful in revealing the downstream lesion anatomy (14); and persistently poor blood flow that failed to allow adequate visualization of the lesion was scored as a total occlusive one. The total score is the sum of individual lesion scores. The CatLet score calculator is available at *www.catletscore.com.*

### Follow-ups

Phone interviews were conducted for all living patients or their immediate relatives with specific questions about major adverse cardiac events (MACEs). All patients were interviewed until the date of the MACEs or the end of this study (September 2019), whichever came first. Medical records, discharge summaries, and angiographic data were systematically reviewed for a patient with an adverse event. Death information was obtained from household registration management systems, hospitals, or the next of kin.

### Endpoints and definitions

The primary endpoint was MACEs at a median of 4.9 years, which was defined as a composite of myocardial infarction, cardiac death, and ischemia-driven repeat revascularization. Secondary endpoints were myocardial infarction, all-cause death, cardiac death, and ischemia-driven repeat revascularization. All-cause death was defined as death from any cause. Deaths were considered cardiac unless a non-cardiac cause could be identified (15). AMI was defined according to the third universal definition of myocardial infarction (16). Ischemia-driven repeat revascularization was defined as revascularization due to clinical ischemic symptoms with a diameter stenosis ≥ 50% or ≥ 70% even in the absence of ischemic symptoms. Clinical factors were defined according to our previous study (17).

### Statistical analysis

Continuous variables were expressed as median (inter-quartile range, IQR). Categorical variables were expressed as frequencies (percentages). Testing for trends in event rates across the tertiles was completed with the STATA procedures opartchi. Missing values were filled in using the multiple imputation methods. The Kaplan–Meier method was employed to generate event-free survival curves, and survival between groups was compared using the trend test. A Cox regression survival analysis was performed to identify the independent predictors of clinical outcomes. ROC curves and calibration plots were used to evaluate the performance of the models. Sensitivity analysis was performed by excluding patients, 1 at a time, with missing values of left ventricular ejection fraction, serum creatinine, and serum albumin. The interaction between the CatLet score on a continuous scale and other risk factors was examined by the *z* test (18). All analyses were performed using Stata version 15.1. All *p*-values and confidence intervals were two-sided.

## Results

A total of 1,212 patients were consecutively enrolled for potential analysis. From the original data set, 1 was excluded for poor images, 5 for coronary embolism, 7 for abnormal coronary anatomy, 140 for normal CAG results, and 41 for loss to follow-up. As a result, all 1,018 patients were finally included in the current study.

### Clinical data and angiographic characteristics

The CatLet score ranged from 2 to 47.5, with a median of 14 (interquartile range, IQR, 10–21). Patients were divided into three groups according to the CatLet tertiles (CatLet_low ≤ 12, CatLet_mid 13–18, and CatLet_top ≥ 19). Clinical data and angiographic characteristics are shown in Table 1 and in online Supplementary Table 1.

**TABLE 1:** Baseline clinical and angiographic characteristics.

Factors	Missing	CatLet_low (≤ 12)	CatLet_mid (13–18)	CatLet_top (≥ 19)	P for trend
N		372	313	333	
Age, years		63.00 (17.50)	65.00 (18.00)	69.00 (14.00)	< 0.01
Gender					0.13
Female		67 (18.01)	74 (23.64)	75 (22.52)	
Male		305 (81.99)	239 (76.36)	258 (77.48)	
Height, cm	2.06%	167.00 (10.00)	167.00 (10.00)	165.00 (10.00)	0.01
Weight, kg	13.56%	66.00 (14.30)	65.00 (12.00)	65.00 (10.00)	< 0.01
STsegment					0.47
STEMI		182 (48.92)	173 (55.27)	153 (45.95)	
non-STEMI		190 (51.08)	140 (44.73)	180 (54.05)	
Length of stent, mm		18.50 (10.50)	20.00 (8.00)	28.00 (29.00)	< 0.01
Hypertension		221 (59.41)	207 (66.13)	245 (73.57)	< 0.01
Diabetes		70 (18.82)	72 (23.00)	94 (28.23)	< 0.01
Smoking					0.04
Never		131 (35.22)	122 (38.98)	130 (39.04)	
Past		26 (6.99)	31 (9.90)	44 (13.21)	
Current		215 (57.80)	160 (51.12)	159 (47.75)	
Alcohol consumption					< 0.01
Never		258 (69.35)	230 (73.48)	263 (78.98)	
Past		10 (2.69)	12 (3.83)	14 (4.20)	
Current		104 (27.96)	71 (22.68)	56 (16.82)	
Diagonal size					0.05
Inter.		259 (69.62)	235 (75.08)	226 (67.87)	
Large		70 (18.82)	54 (17.25)	79 (23.72)	
Small		43 (11.56)	24 (7.67)	28 (8.41)	
LDL-c, mmol/L	1.28%	2.44 (0.93)	2.52 (1.03)	2.44 (1.19)	0.89
HDL-c, mmol/L	1.28%	0.96 (0.21)	0.97 (0.21)	0.93 (0.20)	0.06
TG, mmol/L	1.08%	1.24 (1.00)	1.27 (0.77)	1.20 (0.85)	0.43
TC, mmol/L	1.08%	3.99 (1.26)	4.01 (1.25)	3.96 (1.63)	1.00
Albumin, g/L	1.08%	39.30 (5.15)	38.80 (5.40)	37.10 (6.10)	< 0.01
Lp(a), mg/L	6.88%	102.00 (149.00)	121.00 (189.00)	138.00 (207.00)	0.01
Troponin I, pg/ml	0.59%	6.60 (13.91)	7.05 (36.48)	6.49 (23.66)	0.68
Cr, μmol/L	0.69%	71.00 (22.65)	71.00 (24.00)	73.00 (24.30)	0.69
Blood glucose, mmol/L	0.79%	5.28 (1.37)	5.43 (1.45)	5.46 (1.96)	0.02
LVEF	5.30%	0.56 (0.17)	0.51 (0.18)	0.48 (0.16)	< 0.01

Data were expressed as *n* (%) and median (interquartile range) for categorical and continuous variables, respectively. CatLet_low, the lowest tertile of CatLet score; CatLet_mid, middle tertile; CatLet_top, top tertile; STEMI, ST-segment elevation myocardial infarction; LVEF, left ventricular ejection fraction; LDL-c, low density lipoprotein cholesterol; HDL-c, high density lipoprotein cholesterol; TG, triglycerides; TC, total cholesterol; Lp(a), lipoprotein(a); Cr, creatinine; and LVEF, left ventricular ejection fraction.

### CatLet score and its associations with 5-year outcomes

**FIGURE 1:**
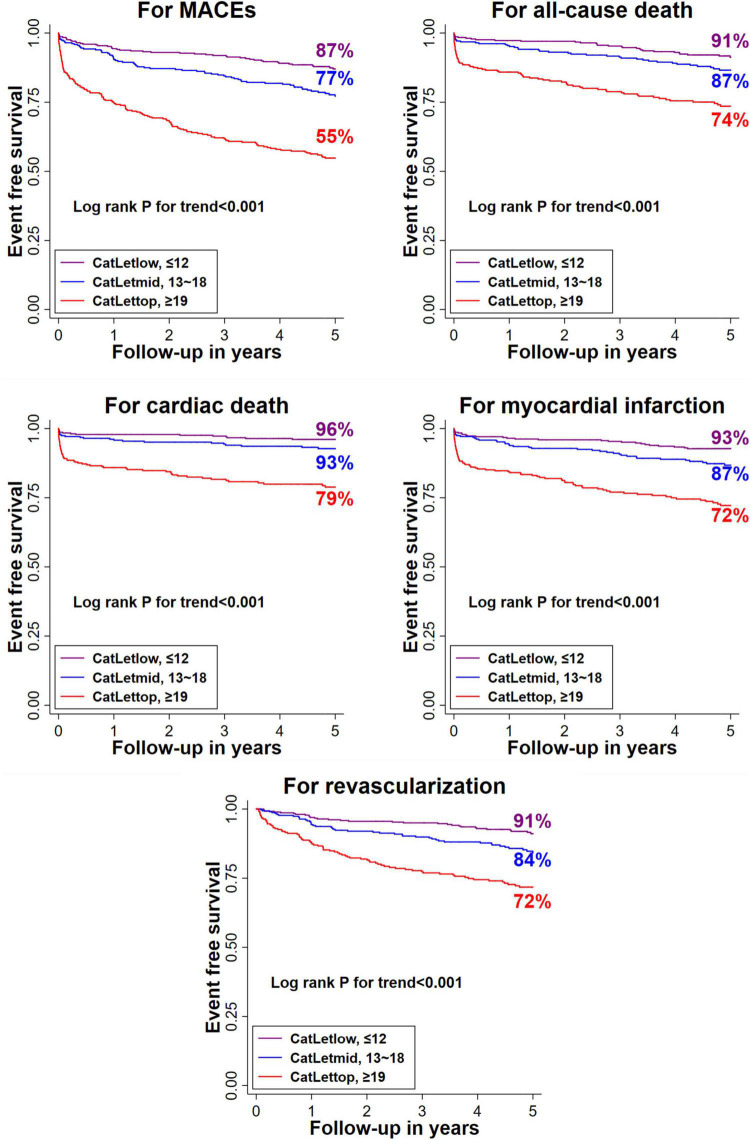
Kaplan–Meier curves for clinical outcomes at 5 years according to the CatLet score tertiles. MACE, major adverse cardiac events.

Figure 1 illustrates that risk stratification was overall balanced between the CatLet score tertiles. The CatLet score predicted clinical outcomes significantly on a continuous or categorical scale as shown in Table 2. After adjustment for age, serum creatinine, LVEF, the CatLet score remains an independent predictor of long-term clinical outcomes. Multivariable-adjusted hazard ratios (95% CI)/unit higher score were 1.06 (1.05–1.08) for MACEs, 1.05 (1.03–1.07) for all-cause death, 1.06 (1.04–1.09) for cardiac death, 1.06 (1.04–1.08) for myocardial infarction, and 1.06 (1.04–1.08) for revascularization. See details in online Supplementary Table 2.

**TABLE 2:** Univariable or multivariable-adjusted HR (95% CI) for higher CatLet score with respect to clinical outcomes on a categorical or continuous scale.

	Crude HR (95% CI)					Adjusted HR (95% CI)				
	Continuous scale	Categorical scale				Continuous scale	Categorical scale			
Outcomes	Per unit CS higher	CS_low (≤ 12)	CS_mid (13–18)	CS_top (≥ 19)	*p* for trend	Per unit CS higher	CS_low (≤ 12)	CS_mid (13–18)	CS_top (≥ 19)	*p* for trend
MACEs	1.08 (1.06–1.09)	Reference	1.84 (1.27–2.68)	4.55 (3.27–6.34)	< 0.001	1.06 (1.05–1.08)	Reference	1.68 (1.15–2.44)	3.59 (2.56–5.03)	< 0.001
All-cause death	1.08 (1.06–1.10)	Reference	1.58 (0.98–2.55)	3.63 (2.38–5.54)	< 0.001	1.05 (1.03–1.07)	Reference	1.26 (0.78–2.04)	2.23 (1.45–3.43)	< 0.001
Cardiac death	1.10 (1.08–1.12)	Reference	1.85 (0.94–3.63)	6.01 (3.36–10.73)	< 0.001	1.06 (1.04–1.09)	Reference	1.39 (0.70–2.74)	3.29 (1.83–5.91)	< 0.001
Myocardial infarction	1.08 (1.06–1.10)	Reference	1.82 (1.10–2.99)	4.39 (2.82–6.83)	< 0.001	1.06 (1.04–1.08)	Reference	1.56 (0.95–2.58)	3.06 (1.95–4.80)	< 0.001
Revascularization	1.06 (1.04–1.08)	Reference	1.84 (1.16–2.93)	3.80 (2.49–5.79)	< 0.001	1.06 (1.04–1.08)	Reference	1.84 (1.16–2.94)	3.83 (2.49–5.89)	< 0.001

Adjusting for age, serum creatinine, left ventricular ejection fraction, and serum albumin. CS, CatLet score; MACE, major adverse cardiac events; and HR, hazard ratio.

### Discrimination

**FIGURE 2:**
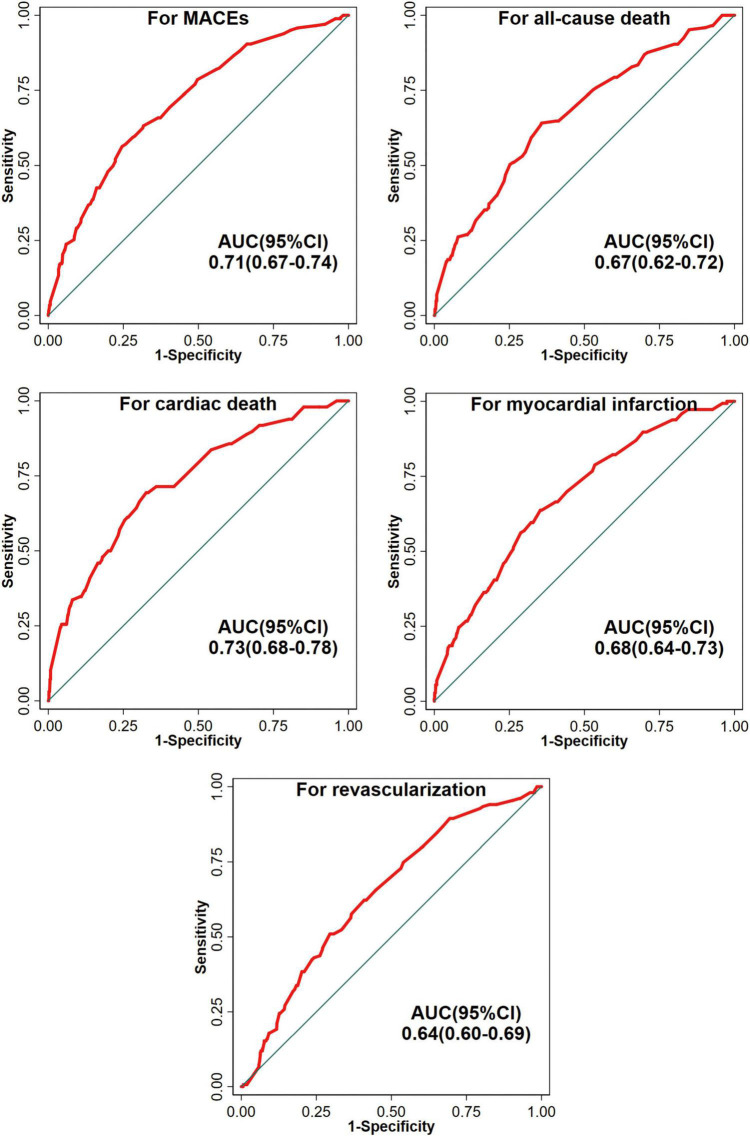
The ROC curves of univariate models for clinical outcomes. MACE, major adverse cardiac events; AUC, Receiver Operating Characteristic (ROC) curve; and CI, confidence interval.

The CatLet score alone performed well in discrimination with respect to clinical outcomes as shown in Figure 2. Areas under the receiver operating characteristic (ROC) curve ranged between 0.6421 and 0.728.

### Calibration

**FIGURE 3:**
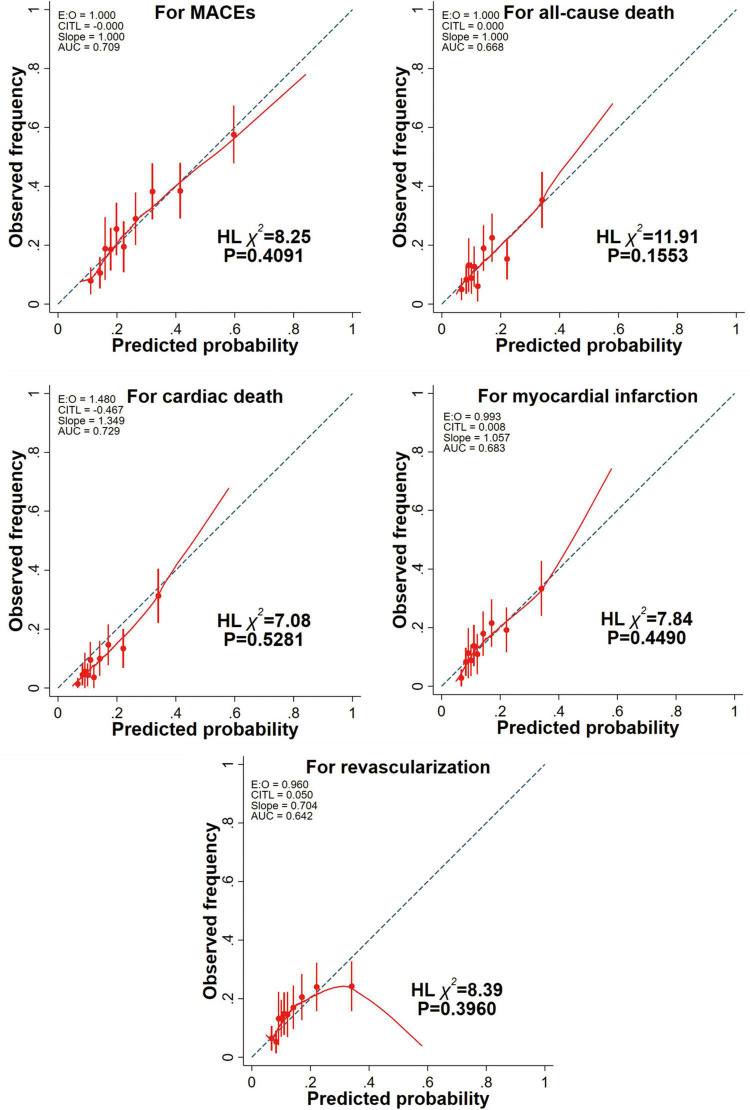
Calibration plots of univariate models for clinical outcomes. A red lowess smoothing curve was added to each calibration plot. Intercept of 0 and slope of 1 indicate perfect prediction. Negative and positive intercepts indicate overestimation and underestimation, respectively. MACE, major adverse cardiac events; HL, Hosmer-Lemeshow test.

In terms of calibration, the model with the CatLet score was robust as shown in Figure 3.

### Subgroup/sensitivity analysis

**FIGURE 4:**
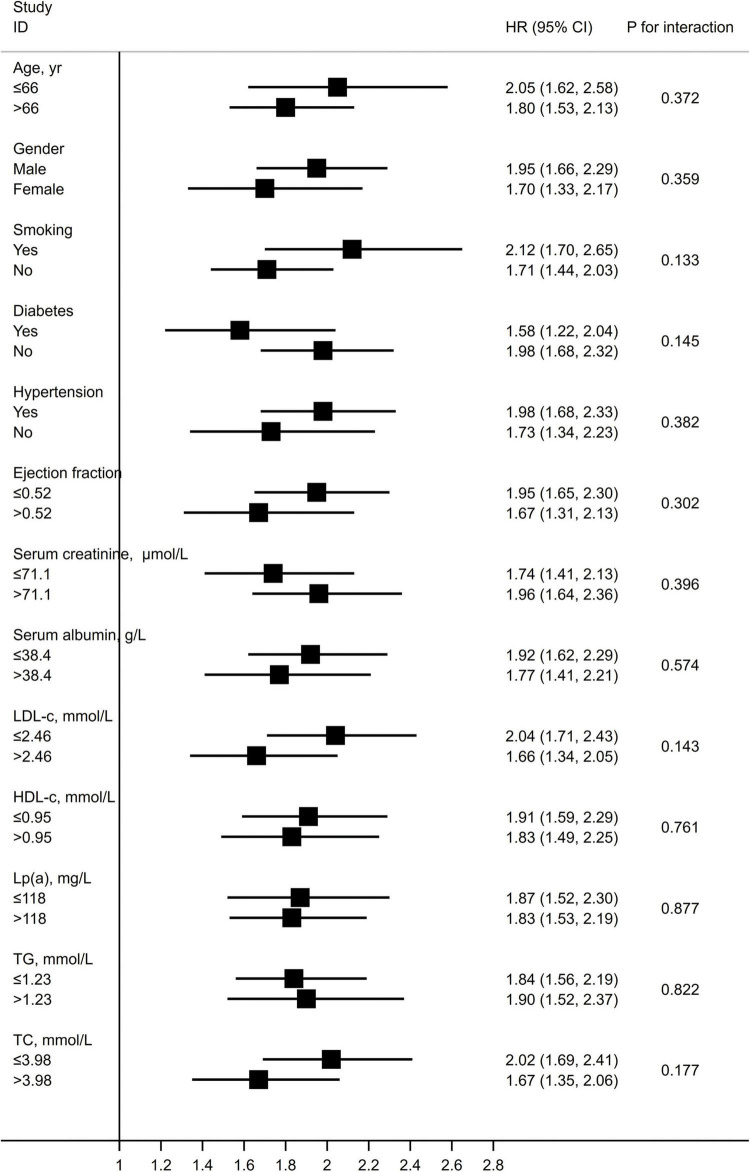
Hazard ratios for MACEs per 10 units higher CatLet score stratified by risk factors, categorically or medially. LDL-c, low density lipoprotein cholesterol; HDL-c, high density lipoprotein cholesterol; Lp(a), lipoprotein(a); TG, triglycerides; and TC, total cholesterol.

Subgroup/sensitivity analysis revealed the consistent associations of the CatLet score with the clinical outcomes, without significant interactions across subgroups as shown in Figure 4 and in online Supplementary Table 2.

## Discussion

The main findings in the current study are as follows: in patients with AMI presenting > 12 h after symptom onset, (a) the CatLet score in isolation independently predicted the 5-year clinical outcomes; (b) the CatLet score remained significantly predictive of 5-year clinical outcomes even after adjustment for important clinical explaining variables, and (c) models with the CatLet score had a robust calibration with respect to clinical outcomes in patients with AMI.

Expectedly, patients with higher CatLet score tertiles were older, had higher rates of diabetes, and had lower serum albumin levels and LVEF, which was consistent with our previous studies (11, 12). Interestingly, in the current study, current smokers had lower CatLet scores, namely “smoker’s paradox.” A similar phenomenon also appeared in our previous studies and some studies of the SYNTAX score (11, 12, 14, 19). Possible explanations include the younger age and fewer cardiovascular risk factors in smokers compared with non-smokers (20). In the current study, patients in the higher CatLet tertiles are younger than those in the lower ones, and age is the strongest predictor of clinical outcomes. Thus, the “smoker’s paradox” appearing in this study can be explained. This association of smoking with clinical outcomes also disappeared after adjusting for covariates (Supplementary Table 2).

In the current study, the AUCs for the CatLet score alone were 0.7289 and 0.6677 for cardiac death and all-cause death, respectively. For the SYNTAX score, another anatomic scoring tool, a meta-analysis including 26 studies indicated that the pooled C-statistics for 1- and 5-year all-cause deaths were 0.65 and 0.62, respectively (21). The performance of the CatLet score is overall better than the SYNTAX score with respect to mortality prediction. These results are wholly anticipated considering that (a) the CatLet score has adequately accounted for the coronary artery trees in its diversity while the SYNTAX score, solely based on the dichotomized right or left coronary dominance, and (b) the CatLet score has simultaneously reflected two aspects of a lesion: the stenosis degree and the myocardial territory (weighting) subtended by the stenotic coronary artery. Our head-to-head comparative study has also shown that the CatLet score had better discrimination and calibration than the SYNTAX score in terms of 4.3-year mortality prediction [C-index, 0.73 (95% CI, 0.66–0.79) vs. 0.69 (0.61–0.77)] (11).

The good discriminatory capacity of the CatLet score between the lower two tertiles as demonstrated in our previous study has been reproduced in the current study (11). By contrast, the SYNTAX score was poor in discriminating between the lower two tertiles (11, 22). Furthermore, large-scale studies on the SYNTAX score did not eliminate this phenomenon (23). We think that it is related to the inherent fallacies of the SYNTAX score with its failure to account for the variable coronary anatomy (11).

Multivariate analyses have been suspected of producing problematic results if fewer outcome events are available relative to the number of independent variables analyzed in the model (24). Therefore, backward stepwise cox regression has been applied to select variables in the current study. Age, serum creatinine, LVEF, and serum albumin were previously reported to be strong predictors of prognosis, and were all finally retained in the multivariate model as well as the CatLet score (Supplementary Table 3) (25–28). Subgroup/sensitivity analysis also did not alter the status of the CatLet score as an independent predictor of clinical outcomes.

## Limitations

There are several limitations to the current study. First, the current study is an observational one in design. Therefore, the findings revealed in this study should be considered as hypothesis-generating. Second, this is a single-centered study enrolling only AMI patients presenting > 12 h after symptom onset. Therefore, our findings warrant further confirmation from different centers and in different CAD populations. Third, like the SYNTAX score, lesions with diameter stenosis ≥ 50% were scored in the CatLet score. However, only 35% of the intermediate (50–70%) angiographic stenosis was hemodynamically relevant as defined by fractional flow reserve (FFR) ≤ 0.80 (29). Further studies are needed to clarify whether the FFR-guided CatLet score is a better predictor of prognosis than the one just based on the visual assessment. Finally, our previous study has shown that the clinically adjusted CatLet score model has a better prognostic value in AMI patients receiving primary PCI (12). In the current study, we also found that clinical variables can in part explain clinical outcomes. Therefore, the effects of clinical variables on the prognostic value of the CatLet score remain to be clarified in AMI patients presenting > 12 h after symptom onset.

## Conclusion

The CatLet score is an independent predictor of long-term prognosis in patients with AMI presenting > 12 h after symptom onset. Our study has extended the application value of the CatLet angiographic scoring system to different AMI populations.

## Data availability statement

The original contributions presented in this study are included in the article/Supplementary material, further inquiries can be directed to the corresponding author.

## Ethics statement

This study was reviewed and approved by the Institute Review Board of Soochow University.

## Author contributions

All authors listed have made a substantial, direct, and intellectual contribution to the work, and approved it for publication.
