# Supplementary material for: The predictive value of CatLet© angiographic scoring system for long-term prognosis in patients with acute myocardial infarction presenting > 12 h after symptom onset
Source: Front Cardiovasc Med. 2022 Sep 21;9:943229. doi: 10.3389/fcvm.2022.943229 (PMC9532528; doi:10.3389/fcvm.2022.943229)
Supplement: Supplementary file 1 [file Data_Sheet_1.docx]

**SUPPLEMENTARY MATERIALS**

**TABLE OF CONTENTS**

**Table S1. Angiographic data for study subjects.**

**Table S2. Hazard ratios of MACEs per 1 unit higher of the variables in multivariate regression**

**Table S3. Sensitivity analysis**

**Table S1. Angiographic data for study subjects.**

| **Factors** | **Missing** | **CatLet_low**  **(≤12)** | **CatLet_mid**  **(13-18)** | **CatLet_top**  **(≥19)** | **P for trend** |
| --- | --- | --- | --- | --- | --- |
| LAD length, n(%) |  |  |  |  | 0.61 |
| Average |  | 203 (54.57) | 189 (60.38) | 208 (62.46) |  |
| Long |  | 113 (30.38) | 89 (28.43) | 85 (25.53) |  |
| Short |  | 56 (15.05) | 35 (11.18) | 40 (12.01) |  |
| RCA dominance, n(%) |  |  |  |  | <0.01 |
| Average RCA |  | 153 (41.13) | 76 (24.28) | 107 (32.13) |  |
| Large RCA |  | 60 (16.13) | 100 (31.95) | 96 (28.83) |  |
| PDA Zero |  | 16 (4.30) | 20 (6.39) | 16 (4.80) |  |
| PDA only |  | 22 (5.91) | 21 (6.71) | 21 (6.31) |  |
| Small RCA |  | 108 (29.03) | 74 (23.64) | 74 (22.22) |  |
| Super RCA |  | 13 (3.49) | 22 (7.03) | 19 (5.71) |  |
| No. of calcification |  | 0.00 (0.00) | 0.00 (0.00) | 0.00 (0.00) | <0.01 |
| No. of lesion |  | 1.00 (0.00) | 1.00 (1.00) | 2.00 (1.00) | <0.01 |
| Culprit vessels, n(%) |  |  |  |  |  |
| LM |  | 0 (0.00) | 1 (0.32) | 30 (9.01) | <0.01 |
| LAD |  | 201 (54.03) | 186 (59.42) | 166 (49.85) | 0.30 |
| LCX |  | 84 (22.58) | 43 (13.74) | 72 (21.62) | 0.68 |
| RCA |  | 90 (24.19) | 87 (27.80) | 117 (35.14) | <0.01 |
| No. of lesion length > 20 mm |  | 23 (6.18) | 33 (10.54) | 99 (29.73) | <0.01 |
| Aorta ostial lesion, n(%) |  | 4 (1.08) | 4 (1.28) | 39 (11.71) | <0.01 |
| Tortuosity, n(%) |  | 119 (31.99) | 118 (37.70) | 214 (64.26) | <0.01 |
| Thrombus, n(%) |  | 212 (56.99) | 167 (53.35) | 175 (52.55) | 0.23 |
| Angulation < 70, n(%) |  | 79 (21.24) | 87 (27.80) | 137 (41.14) | <0.01 |
| Trifurcation, n(%) |  | 3 (0.81) | 0 (0.00) | 0 (0.00) | 0.05 |
| No. of bifurcation, n(%) |  |  |  |  |  |
| Medina 0,0,1 |  | 14 (3.76) | 23 (7.35) | 37 (11.11) | <0.01 |
| Medina 0,1,0 |  | 58 (15.59) | 50 (15.97) | 82 (24.62) | <0.01 |
| Medina 0,1,1 |  | 4 (1.08) | 5 (1.60) | 22 (6.61) | <0.01 |
| Medina 1,0,0 |  | 33 (8.87) | 50 (15.97) | 62 (18.62) | <0.01 |
| Medina 1,0,1 |  | 2 (0.54) | 2 (0.64) | 4 (1.20) | 0.33 |
| Medina 1,1,0 |  | 5 (1.34) | 8 (2.56) | 24 (7.21) | <0.01 |
| Medina 1,1,1 |  | 9 (2.42) | 8 (2.56) | 35 (10.51) | <0.01 |
| Treated coronary artery, n(%) |  |  |  |  |  |
| LCX |  | 76 (20.43) | 42 (13.42) | 78 (23.42) | 0.36 |
| LAD |  | 190 (51.08) | 186 (59.42) | 168 (50.45) | 0.93 |
| LM |  | 0 (0.00) | 1 (0.32) | 28 (8.41) | <0.01 |
| RCA |  | 92 (24.73) | 94 (30.03) | 146 (43.84) | <0.01 |
| No. of diseased vessels, n(%) |  |  |  |  | <0.01 |
| 0 |  | 0 (0.00) | 1 (0.32) | 10 (3.00) |  |
| 1 |  | 347 (93.28) | 210 (67.09) | 29 (8.71) |  |
| 2 |  | 25 (6.72) | 97 (30.99) | 183 (54.95) |  |
| 3 |  | 0 (0.00) | 5 (1.60) | 111 (33.33) |  |
| Diseased LM, n(%) |  | 0 (0.00) | 1 (0.32) | 49 (14.71) | <0.01 |

LCX=left circumflex; LAD=left anterior descending artery; LM=left main; RCA=right coronary artery;

**Table S2.** Hazard ratios of MACEs per 1 unit higher of the variables in multivariate regression

| Variables | HR | SE | z | *P* | 95%CI |
| --- | --- | --- | --- | --- | --- |
| CS | 1.06 | 0.01 | 9.05 | <0.001 | 1.05-1.08 |
| age, year | 1.02 | 0.01 | 2.84 | 0.005 | 1.01-1.03 |
| Cr, μmol/dL | 1.02 | 0.01 | 2.64 | 0.008 | 1.01-1.04 |
| LVEF, % | 0.12 | 0.07 | -3.42 | 0.001 | 0.04-0.41 |
| Albumin, g/L | 0.95 | 0.01 | -3.54 | <0.001 | 0.93-0.98 |

Abbreviations: CS=the CatLet score; Cr=serum creatinine; LVEF=left ventricular ejection fraction; HR= hazard ratios; SE=standard error; and CI=confident interval.

**Table S3.** Sensitivity analysis

| Endpoints | HR1(95%CI) | HR2(95%CI) | HR3(95%CI) |
| --- | --- | --- | --- |
| MACEs | 1.06(1.05-1.08) | 1.06(1.05-1.08) | 1.06(1.05-1.08) |
| All-cause death | 1.04(1.03-1.07) | 1.05(1.03-1.07) | 1.05(1.03-1.07) |
| Cardiac death | 1.06(1.04-1.08) | 1.06(1.04-1.09) | 1.06(1.04-1.09) |
| MI | 1.06(1.04-1.08) | 1.06(1.04-1.08) | 1.06(1.04-1.08) |
| Revascularization | 1.06(1.04-1.08) | 1.06(1.04-1.08) | 1.06(1.04-1.08) |

Note: HR1, 2, and 3 indicated hazard ratios of the CatLet score in multivariate model after omitting the missing values one by one of left ventricular ejection fraction, serum creatinine, and serum albumin, respectively.
